# Mannitol for prevention of acute kidney injury after liver transplantation: a randomized controlled trial

**DOI:** 10.1186/s12871-022-01936-7

**Published:** 2022-12-19

**Authors:** Moataz Maher Emara, Doaa Galal Diab, Amr Mohamed Yassen, Maha A. Abo-Zeid

**Affiliations:** 1 Department of Anesthesiology and Intensive Care and Pain Medicine, Mansoura University, Faculty of Medicine, Mansoura, Egypt; 2 Liver Transplantation program, Mansoura University, Gastrointestinal Surgery Center, Mansoura, Egypt

**Keywords:** Mannitol, Acute kidney injury, Creatinine, Living donor, Liver transplantation, Injury, Ischemia reperfusion, Randomized controlled trial

## Abstract

**Background:**

Acute kidney injury (AKI) is a common complication after liver transplantation, which is associated with increased morbidity and mortality. Therefore, this study investigated mannitol as an oxygen-free radical scavenger and its role in the prevention of early AKI after living donor liver transplantation (LDLT).

**Methods:**

A total of 84 adult patients who underwent LDLT were randomly assigned to two equal groups: the M group, where patients received 1 g/kg mannitol 20%, or the S group, where patients received an equal volume of saline. The primary outcome was the incidence of early AKI, defined as a 0.3 mg/dl increase in the serum creatinine 48 h postoperatively. Laboratory assessments of the graft and creatinine were recorded until 3 months after transplantation besides the post-reperfusion syndrome and the intraoperative hemodynamic measurements.

**Results:**

The AKI incidence was comparable between groups (relative risk ratio of 1.285, 95% CI 0.598–2.759, *P* = 0.518). Moreover, AKI stages and serum creatinine 3 months after transplantation, *P* = 0.23 and *P* = 0.25, respectively. The incidence of the post-reperfusion syndrome was comparable in both groups, 29/39 (74.4%) and 31/41 (75.6%) in M and S groups, respectively, *P* = 0.897. The intraoperative hemodynamic parameters showed no significant difference between groups using the area under the curve.

**Conclusion:**

The current LDLT recipient sample was insufficient to demonstrate that pre-reperfusion 1 g/kg mannitol infusion would reduce the risk of early AKI or post-reperfusion syndrome.

**Clinical trial registration number:**

Pan African Clinical Trials Registry (PACTR202203622900599); https://pactr.samrc.ac.za/TrialDisplay.aspx?TrialID=21511.

**Supplementary Information:**

The online version contains supplementary material available at 10.1186/s12871-022-01936-7.

## Introduction

Liver transplantation became the standard treatment for end-stage liver disease and selected cases of liver neoplasms [1]. The incidence of acute kidney injury (AKI) can reach up to 95% after the liver transplantation (LT) setting due to ischemia-reperfusion injury (IRI) [2].

Degradation of adenosine triphosphate (ATP) molecules results in an accumulation of hypoxanthine during liver graft ischemia. Hypoxanthine produces toxic reactive oxygen species (ROSs) by xanthine oxidase after reperfusion and reoxygenation of the graft [2, 3]. Those ROS produce cellular injury by lipid peroxidation of the cell membranes, leukocyte activation, chemotaxis, and leukocytes-endothelial adhesion [2, 3].

Mannitol, vitamin C, vitamin E, superoxide dismutase, N-acetyl cysteine, and allopurinol are common examples of antioxidants [4]. Mannitol and ascorbic acid were effective in scavenging and inhibiting ROS after liver IRI on the histopathological and biochemical levels [5]. Additionally, mannitol creates a hyperosmolar environment, which may blunt the IRI [6].

Mannitol drip within 15 min of cross-clamping enhances renal preservation during living donor kidney transplantation [7]. While in LT, mannitol infusion during the anhepatic phase could ameliorate post-reperfusion syndrome (PRS) [8], Whitta and colleagues found no protective effect of mannitol on kidney function during LT [9].

We hypothesized that using mannitol during the anhepatic phase in living donor liver transplantation (LDLT) would decrease the early AKI incidence. The aim of the study was to investigate the role of mannitol for prevention of AKI and PRS in LDLT.

## Patients and methods

This randomized controlled study was conducted on adult (≥ 18 years old) patients of either sex, who underwent LDLT with right lobe graft from family-related donors between 10 December 2017 and 22 October 2019 at the Gastrointestinal Surgery Center, Mansoura University, Egypt. Institutional review board approval was obtained (MD/17.08.28) on 7 September 2017 and all methods were performed in accordance with declaration of Helsinki. The study was registered at the Pan African Clinical Trials Registry (PACTR202203622900599) on 23 March 2022. All the included patients were granted informed consent. This study was reported according to the CONsolidated Standards of Reporting Trials (CONSORT) guidelines [10].

Exclusion criteria were acute fulminant hepatitis, estimated graft/recipient weight ratio (GRWR) <0.8, and portal hypertension with mean pulmonary blood pressure >35 mmHg. Patients with preoperative S.Cr >1.4 mg/dL or had dialysis within the last 3 months, diabetes mellitus (>10 years), preoperative serum sodium [Na+] <125 mEq/L, or serum potassium [K+] >5.5 mEq/L were also excluded.

### Randomization and blinding

A total of 84 patients were randomly assigned to two equal groups: mannitol group (M group) and saline group (**S** group) using a computer-generated table of random numbers with four or six permuted blocks. The group allocation was concealed in sequentially numbered, sealed, and opaque envelopes.

An anesthetist—who was not involved in the study—opened the envelopes before the end of the dissection phase. He then prepared the study drug as indicated in the envelope using a similar volume of either 1 g/kg mannitol 20% or saline 0.9% in a similar warmed unlabeled bottle wrapped by an opaque cover.

The patients, outcome assessors, and the statistician were blinded to the study group until the results were reported.

### Patients’ preparation and anesthesia

All patients fasted for 6 h preoperatively for solids and were encouraged to freely drink water up to 4 h preoperatively with an infusion of 500 mL Ringer’s acetate during the fasting period.

Baseline recipients’ characteristics and laboratory data were collected 24 h before the operation. Donor age and gender were recorded in the study after considering the donors’ data. General anesthesia was induced by intravenous (IV) fentanyl 2 mcg/kg, propofol 1–2 mg/kg, and rocuronium bromide 0.8–1 mg/kg. Anesthesia was maintained by sevoflurane in 40%–60% oxygen in addition to a fentanyl infusion of 0.5–1 mcg/kg/h and rocuronium bromide 200–400 mcg/kg/h. All the patients were kept warm by forced-air warming blankets.

Invasive arterial blood cannula and pulmonary artery catheter were inserted for continuous intraoperative cardiac output (CO) and temperature monitoring (ABBOTT, critical care systems, USA).

Ringer’s acetate was used as the maintenance solution. Our center adopted a goal-directed fluid protocol in LDLT and targeted to maintain the mean arterial pressure (MAP) of ≥ 65 mmHg. Patients with stroke volume variation (SVV) >10% were considered fluid responders and received boluses of 200 mL albumin 4% in Ringer’s acetate. The hemoglobin concentration threshold for red blood cells (RBCs) transfusion was 7–8 g/dL according to the clinical judgment. Norepinephrine infusion was started in fluid non-responders.

Random blood glucose was kept between 110 mg/dL and 180 mg/dL by intravenous insulin infusion or boluses of 10% or 25% glucose solution as appropriate. Also, serum potassium (K+) and ionized calcium (Ca2+) levels were monitored and corrected if needed, especially around the reperfusion phase.

### Mannitol and reperfusion

The graft preservation time was minimized by synchronizing the surgical steps of both recipient and donor operations. It was flushed with 3–4 L of cold histidine-tryptophan-ketoglutarate (Custodial, Bensheim, Germany) via antegrade flushing of the portal vein (without flushing via the hepatic artery) to get completely clear fluid after excision of the donor’s right liver lobe.

The study solution was infused for over 20 min at the beginning of warm ischemia. The right hepatic vein was unclamped, followed by the portal vein, and then the graft preservative contents were washed into the systemic circulation by the portal blood.

### Postreperfusion syndrome and hemodynamic parameters

Hypotension was defined as a 20% reduction below the basal MAP while PRS was defined as a 30% drop in the MAP compared to the basal reading sustained for 1 min within 5 min after portal unclamping.

Both groups were managed by rapid 500 mL 4% albumin infusion or packed RBCs (according to the anhepatic phase hemoglobin) and 20 mcg norepinephrine boluses, and then infusion if needed. Incremental boluses of 10 mcg epinephrine were administered if MAP was still less than 65 mmHg after 1 min.

The intraoperative hemodynamic parameters [MAP, CO, systemic vascular resistance (SVR), pulmonary vascular resistance (PVR), pulmonary artery occlusion pressure (PAOP), mean pulmonary arterial pressure (MPAP), and central venous pressure (CVP)], and serum electrolyte [Na+, K+, Ca2+ and chloride (Cl-)] levels were recorded at six intraoperative measurement points. These moments were (1) immediately before skin incision, (2) at the beginning of the anhepatic phase (portal vein clamping), (3) 5 min before portal reperfusion (basal), (4) at 5 min after portal unclamping, (5) at 5 min after hepatic arterial declamping, and (6) at skin closure.

### At the end of surgery and in the intensive care unit (ICU)

Early ICU tracheal extubation was adopted once the patient was hemodynamically stable [MAP >65 mmHg, heart rate (HR) <100 beats per min, and peripheral oxygen saturation (SpO_2_) >96% on 0.4 fractions of inspired oxygen (FiO2)] and pH >7.3 with adequate consciousness level and muscle power.

Daily zero fluid balance was targeted during the ICU stay. Fluid maintenance was Ringer’s acetate and glucose 10% encouraging early oral fluids from the first postoperative day. Albumin was administered to keep the serum albumin ≥ 3.0 g/dL.

Early AKI (primary outcome) was defined as a 0.3 mg/dl increase in the serum creatinine (S.Cr) in the early 48 postoperative hours, according to the International Club of Ascites' revised classification of AKI in cirrhotic patients [11, 12]. The AKI has been classified as follows: Stage 1, when S.Cr = 1.5–1.9 times at the baseline or >0.3 mg/dl increase from the baseline; Stage 2, when S.Cr = 2–2.9 times at the baseline; and Stage 3, when S.Cr = 3 times at the baseline increases to >4 mg/dl or results in the initiation of renal replacement therapy [11, 12].

### Urine output (UO) during surgery and in the ICU

The UO was monitored hourly intraoperatively. Furosemide 5 mg IV was administered when UO is less than 0.5 mL/kg/h after ensuring adequate fluid status. In the ICU, 5–10 mg furosemide was given if UO is less than 0.5 mL/kg/h and evaluated every 6 h if volume overload was estimated.

### Immunosuppression

Patients received IV 0.5 gm methylprednisolone at the beginning of the warm ischemia. A 500 mg mycophenolate mofetil through the nasogastric tube and IV 20 mg basiliximab were administered after hepatic artery anastomosis and declamping.

Patients received oral tacrolimus from the day after the operation (adjusting the dose targeting serum level of 5–10 ng/mL) and mycophenolate mofetil 500 mg 4 days after the operation. Tacrolimus was replaced, temporarily, with methylprednisolone if AKI is diagnosed until normal kidney function was restored.

### Postoperative data

Laboratory assessment of the graft function included pH, serum lactate, and lactate dehydrogenase (LDH) recorded on the 1st and 2nd days, while S.Cr, aspartate aminotransferase (AST), alanine aminotransferase (ALT), bilirubin, albumin, and international normalized ratio (INR) were measured at the 1st, 2nd, 7th, 28th days and also after 3 months postoperatively. The ICU and the duration of hospitalization, early postoperative surgical complications (within 28 days), and 3-month survival after the operation were reported.

## Sample size calculation

A pilot study was performed using the control [saline (S)] group and found a 60% incidence rate of early AKI within 48 h of LDLT. About 39 patients were required in each group to demonstrate an absolute 50% decrease in the incidence rate of AKI after using mannitol assuming α = 0.05 and β = 0.2 (80% power) and using the chi-squared test. A total of 42 patients were required in each group to allow for subject dropout.

## Statistical analysis

The SPSS for Windows software (version 21.0; SPSS Inc, Chicago, Ill, United States) was used to analyze available data from 80 patients (39 cases in the M group and 41 in the S group). Continuous data were tested for normalization purposes using the histogram, Q-Q plot, and the Kolmogorov-Smirnov tests.

Normally distributed data were presented as the mean (standard deviation) and compared using Student’s independent *t*-test. Unevenly distributed data were presented as median (interquartile range) and were compared using the Mann–Whitney U test. We dealt with intraoperative serial measurement data using the area under the curve as advised by Matthews and colleagues [13]. We then compared the groups using the Student’s independent *t*-test.

Categorical data were depicted as numbers (percentage) and compared using the chi-squared test or Fisher’s exact test, as appropriate. A two-tailed *P*-value of <0.05 was considered statistically significant.

## Results

**Fig. 1:**
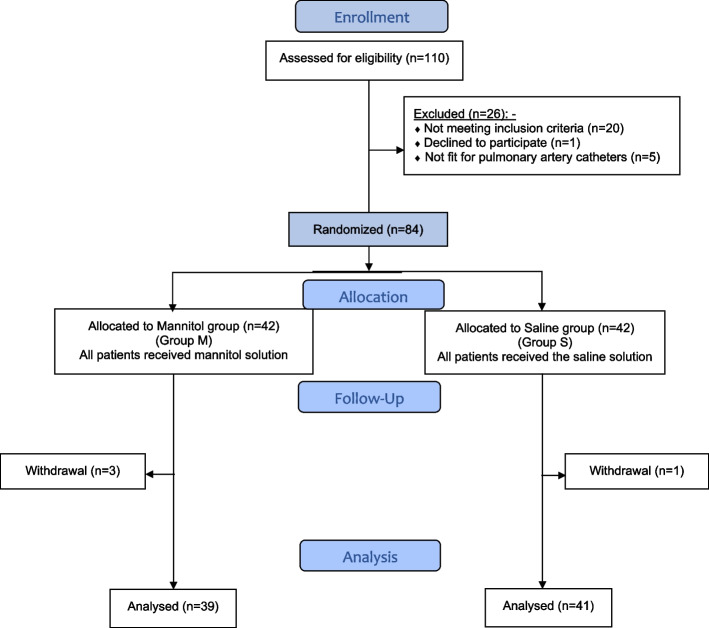
CONSORT Flow Chart

The current study assessed 110 patients for eligibility; 26 patients were excluded. Subsequently, 84 patients assigned for LDLT surgery were randomized to receive either mannitol (Group M) or saline (Group S). Records of three patients in the M group and one patient in the S group were lost in the follow-up. Thus, the data of 39 patients in the M group and 41 patients in the S group were analyzed (Fig. 1).

Recipients’ characteristics and preoperative laboratory data of both groups showed no statistical differences (Table 1).

**Table 1:** Liver transplantation recipients’ baseline characteristics and preoperative laboratory results

	Group (M) *N* = 39	Group S *N* = 41	*P*-Value
Age (years)	53 (43–57)	54 (45–57)	0.858
Sex (Male/Female No., %)	27/12 (69.2/ 30.8%)	29/12 (70.7/39.3%)	0.884
Weight (Kg)	77.62 (10.7)	78.59 (10.9)	0.69
Height (m)	1.68 (0.08)	1.68 (0.07)	0.893
Body Surface Area (m^2^)	1.87 (0.15)	1.88 (0.02)	0.645
MELD score	15 (12–17)	15 (11–17)	0.703
CPC	8 (7–10)	8 (6–10)	0.873
Etiology (No., %)			
Viral hepatitis	19/39 (48.7%)	15/41 (36.6%)	0.445
Hepatocellular carcinoma	12/39 (30.8%)	19/41 (46.3%)	
Autoimmune disease	7/39 (17.9%)	5/41 (12.2%	
Cryptogenic	1/39 (2.6%)	2/41 (4.9%)	
Comorbidities (No., %)			
HTN	0/39	1/41 (2.5%)	0.119
DM	10/39 (25.6%)	11/41 (27.5%)	
DM and HTN	0/39	5/41 (12.5%)	
Cardiac (mild mitral regurgitation)	0/39	1/41 (2.5%)	
Hypothyroidism	1/39 (2.6%)	0/41	
S.Cr. (mg/dL)	0.7 (0.6–0.8)	0.7 (0.6–0.9)	0.221
S. AST (u/mL)	40 (30–60)	48 (39–76)	0.068
S. ALT (u/mL)	21 (21–34)	27 (21–54)	0.35
S. total bilirubin (mg/dL)	2.3 (1.6–3.3)	1.9 (1.55–3.4)	0.765
S. direct bilirubin (mg/dL)	1 (0.6–1.5)	1.1 (0.55–1.8)	0.821
S. albumin (g/dL)	2.9 (0.68)	2.8 (0.53)	0.41
INR	1.5 (0.29)	1.46 (0.29)	0.577
CRP (mg/dL)	0 (0–8)	0 (0–9)	0.808

Data are presented as mean (*SD*) or median (IQR), and No. (%), *SD* standard deviation, *IQR* interquartile range, *M group* mannitol group, *S group* saline group, *MELD* model for end-stage liver disease, *CPC* Child-Pugh classification, *HCC* hepatocellular carcinoma, *HTN* hypertension, *DM* diabetes mellitus, *INR* international normalized ratio, *AST* Aspartate Transaminase, *ALT* Alanine Transaminase, *S* serum, *Cr* Creatinine, *CRP* C-reactive protein

Regarding the donors’ data, the mean age was 27 (6) in the M group and 27 (7) years in the S group (*p*-value = 0.625). There were 27 male donors (69.2%) in the M group and 29 (70.7%) in the **S** group (*p*-value = 0.884).

### AKI incidence and AKI stages

The study’s primary outcome was AKI incidence and was comparable between groups 11/39 (28.2%) in M and 9/41 (22%) in the S groups, respectively, (*P* = 0.518), with a relative risk ratio of 1.285, 95% CI 0.598–2.759.

Differences in AKI stages between groups are not statistically or clinically significant (*P* = 0.225). Zero cases in the M group versus two (4.9%) cases in the S group reached stage III AKI. About 15.4% (6/39) of those who received mannitol reached stage II when compared to 4.9% (2/41) in the S group, while five cases in each group had stage I AKI (12.8% in the M group and 12.2% in the **S** group).

Renal function was comparable in both groups at the 3 months post-LDLT (Supplementary Table 2). No case in the two groups needed dialysis during the study period (3 months post-LDLT) in this study.

### Intraoperative hemodynamics

**Fig. 2:**
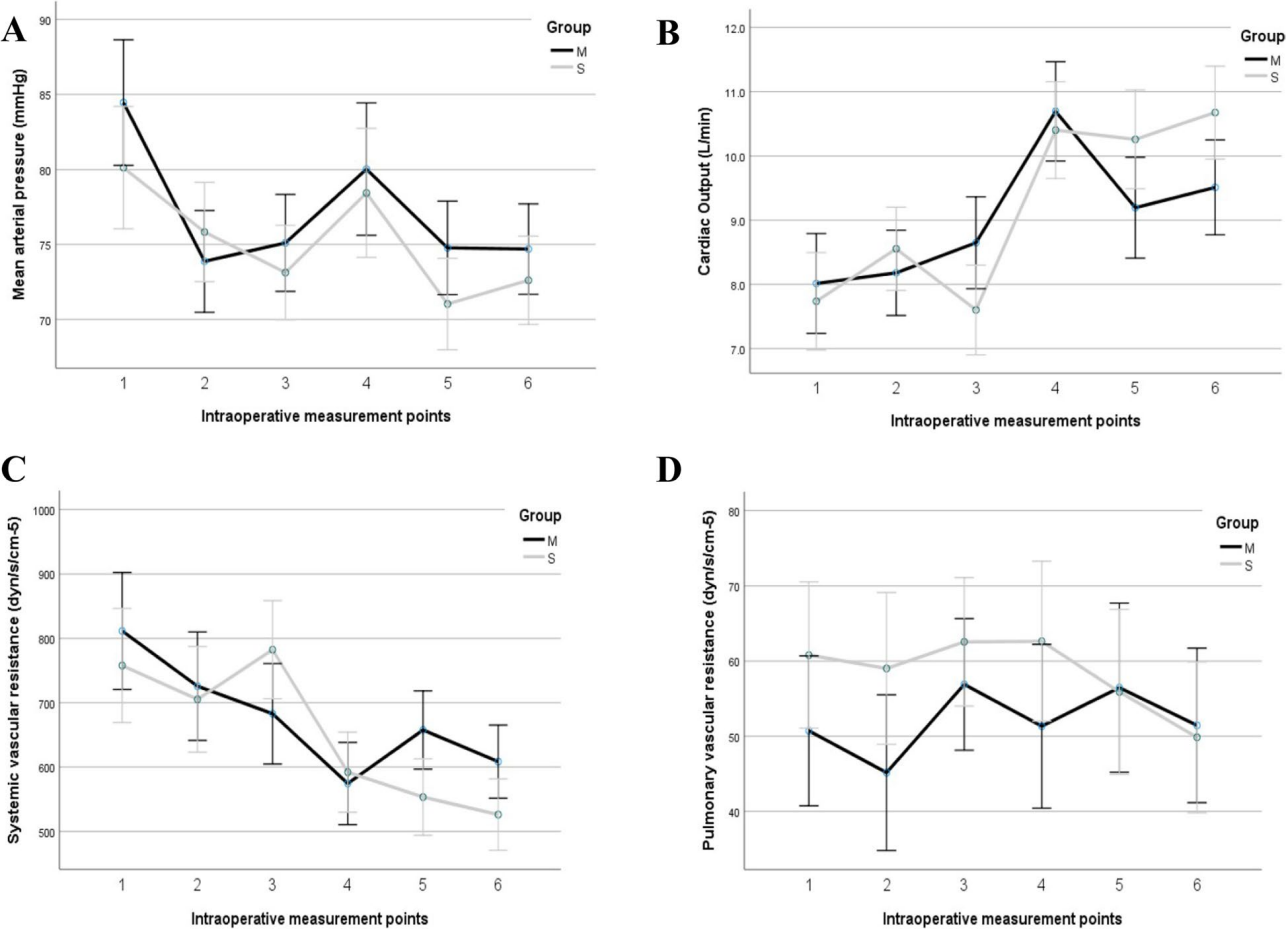
Intraoperative Hemodynamic parameters: **A** MAP = Mean arterial pressure MAP (mmHg);** B** CO = Cardiac Output (L/min); **C** SVR= Systemic vascular resistance (dyn/s/cm^-5^); **D** PVR = Pulmonary vascular resistance (dyn/s/cm^-5^) at 6 intraoperative measurement points: (1) immediately before skin incision, (2) the beginning of the an-hepatic (portal vein clamping), (3) 5-minutes before portal reperfusion (basal), (4) at 5-min after portal unclamping, (5) 5-minutes after hepatic arterial de- clamping and (6) at the skin closure. M group = mannitol group, S group =saline group. Data are presented as mean (SD)

At the six intraoperative measurement points, all intraoperative hemodynamic parameters including MAP, CO, SVR, and PVR in Fig. 2 and PAOP, MPAP, and CVP in Supplementary Figure S1 showed no significant difference between the groups.

### Intraoperative electrolytes

**Fig. 3:**
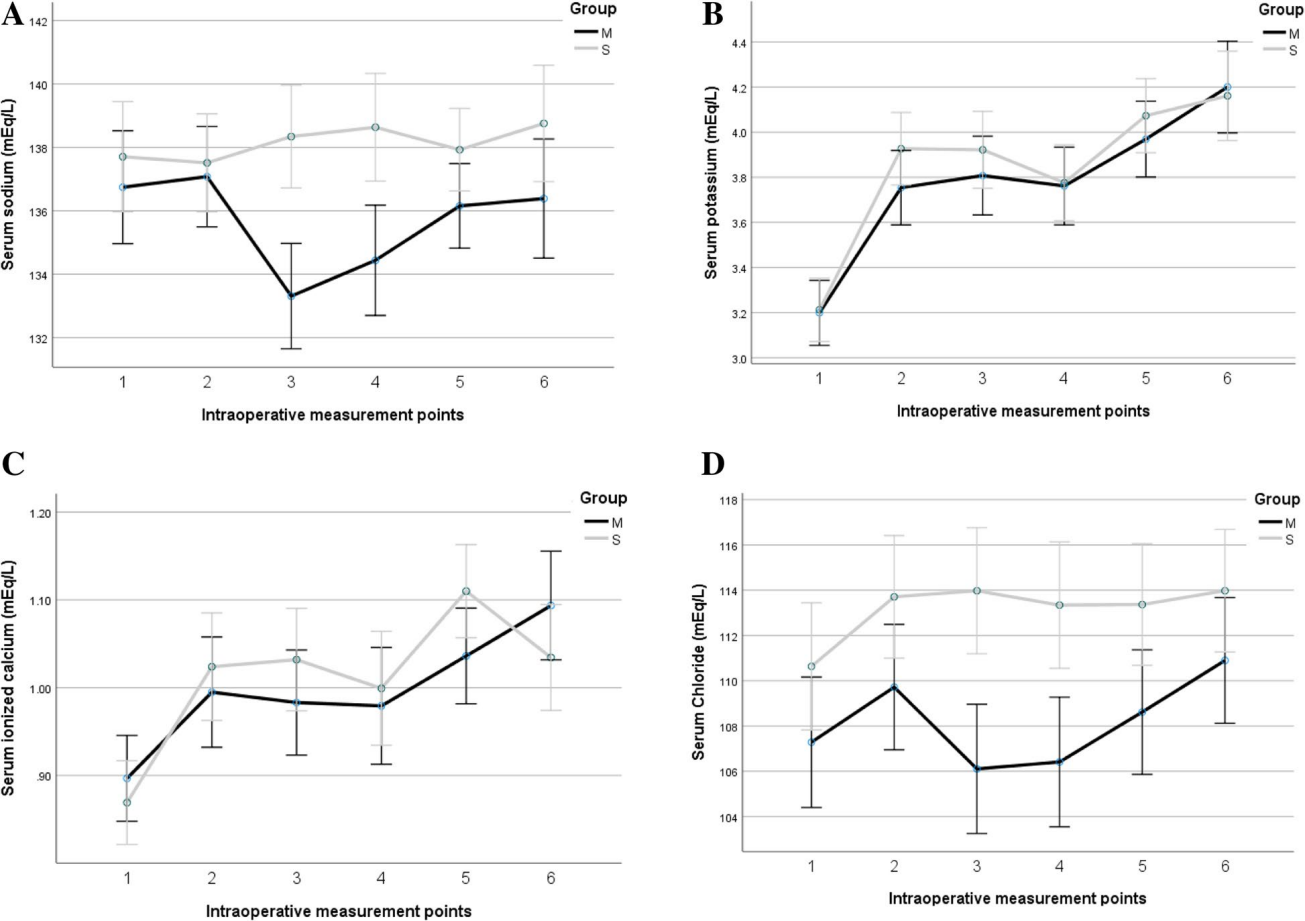
Intraoperative electrolytes: **A** serum Sodium (mEq/L);** B** serum Potassium (mEq/L); **C** serum ionized calcium (mEq/L); **D** serum Chloride (mEq/L) at 6 intraoperative measurement points: (1) immediately before skin incision, (2) the beginning of the an-hepatic (portal vein clamping), (3) 5-minutes before portal reperfusion (basal), (4) at 5 min after portal unclamping, (5) 5-minutes after hepatic arterial de-clamping and (6) at the skin closure. M group = mannitol group, S group = saline group. Data are presented as mean (SD)

Intraoperative serum K+ and ionized Ca+2 did not differ significantly between groups at all the six times of measurements. The average Na+ at 5 min before and after portal reperfusion was significantly higher in the S group (138 and 139 mEq/L) versus (133 and 135 mEq/L) in the M group [95% CI (2.7–7.4) and (1.4–6) mEq/L, respectively]. Moreover, Cl- on average was significantly higher in the S group 5 min before and 5 min after portal declamping, and 5 min after hepatic artery declamping (Fig. 3).

### Other operative data and PRS

The PRS incidence was comparable in both groups, 29/39 (74.4%) and 31/41 (75.6%) in the M and S groups, respectively, *P*-value = 0.897.

Intraoperative UO was significantly higher in the M group compared to the S group during the anhepatic phase (350 (20-500) versus 150 (50-325) mL, respectively; 95% CI 66.5–235 L, *P*-value = 0.001) and the whole operative time 2100 (1340-3350) mL versus 1400 (1025-2425) mL respectively, *P*-value = 0.027; Table 2).

**Table 2:** Intraoperative ischemia and anhepatic phase times, operative duration, intraoperative blood product transfusion, UO, and total doses of furosemide, norepinephrine, and epinephrine

	Group (M) ***N*** = 39	Group (S) ***N*** = 41	***P***-Value
**Cold ischemia** (minutes)	28.7 (10.5)	29.8 (9.9)	0.655
**Warm ischemia** (minutes)	35.8 (8.6)	38.4 (9.2)	0.2
**Anhepatic phase duration** (minutes)	70 (15.6)	73.6 (17.3)	0.348
**Operative duration** (minutes)	567.6 (70.4)	576 (78.2)	0.636
**RBCs** (No. of patients)	24/38 (63.2%)^#^	28/41 (68.3%)	0.644
**Fresh frozen plasma** (No. of patients)	3/38 (7.9%)^#^	5/41 (12.2%)	0.713
**Platelets** (No. of patients)	0/38	0/41	-
**RBCs** (No. of units)	1 (0–3.25)	2 (0–3.5)	0.695
**Plasma** (No. of units)	0 (0–7)	0 (0–5)	0.549
**Anhepatic phase UO** (mL)	350 (200–500)	150 (50–325)	0.003*
**Total UO** (mL)	2100 (1340–3350)	1400 (1025–2425)	0.027*
**Total intraoperative furosemide** (mg)	5 (0–10)	5 (0–10)	0.528
**Total intraoperative norepinephrine** (mcg)	200 (77.5–275)	190 (120–270)	0.97
**Total intraoperative epinephrine** (mcg)	0 (0–25)	0 (0–20)	0.35

Data are presented as mean (*SD*), median, (*IQR*) or No. (%). * Indicates statistical significance (*P*-value <0.05), *M group* mannitol group, *S group* saline group, *UO* urine output, *RBCs* Red blood cells

**#** missed data of one patient

Cold ischemia, warm ischemia, anhepatic phase, and operative duration did not differ significantly between the two groups, as well as intraoperative blood products, transfusion, and total intraoperative consumption of furosemide, norepinephrine, and epinephrine doses **(**Table **2****).**

### Postoperative data

The pH, serum lactate, and LDH in the first two ICU days did not display significant variance between both groups as shown in Supplementary Table 1.

Supplementary Table 2 presents the quite similarities between both groups regarding Cr, AST, ALT, total bilirubin, albumin, and INR from the first postoperative day until 3 months post-transplant, except total bilirubin on the 7th day and albumin after 3 months, which were significantly higher in the M group with a median 4.7 mg/dl and 4.44 g/dL versus 2 and 4.21 in the S group, respectively.

Both groups were similar regarding the mean ICU stay duration, the incidence of postoperative surgical complications, and the 3-month survival period. Those values were 6.08 (1.98) days, 10 (25.6%), and 38 (97.4%) cases in the M group versus 5.54 (2) days, 7 (17.1%) and 37 (90.2%) cases in the S group with *p*-values of 0.228, 0.418, and 0.36, respectively.

## Discussion

This is a triple-blinded randomized controlled study of the role of mannitol in the prevention of AKI in LDLT. The AKI incidence was almost similar between the groups. Most intraoperative hemodynamic parameters did not show statistical differences between the groups. Both Na+ and Cl- were higher in the S group at 5 min before and after portal vein declamping (graft reperfusion). The intraoperative UO was higher in the M group.

Patient characteristics and AKI risk factors including operative time, cold ischemia, warm ischemia, anhepatic time, intraoperative blood transfusion, and the incidence of PRS were comparable in both groups.

Mannitol could not prevent early AKI following LDLT with a tendency toward a higher AKI incidence in the M group—11/39 (28.2%) versus 9/41 (22%) in the S group, respectively (*P*-value = 0.518).

Mannitol did not prevent acute renal failure during LT which is in agreement with a study by Whitta et al. Nonetheless, they only studied 25 patients with 12 cases in the M group [9]. However, they started mannitol infusion after induction of anesthesia, which is a long time before reperfusion while the plasma elimination half-life of mannitol was 2.44 h and the duration of action may extend to 8 h after the end of infusion [14]. Mannitol could not prevent the effects of hepatic ischemic-reperfusion injury on renal function unlike in the biochemical and animal studies [5, 15, 16]. Mannitol could not downstage the AKI even when the AKI occurred, as shown in the existing study. There were no statistical or clinical differences between the groups regarding the AKI stages.

Mannitol did not reduce the incidence of PRS in the current study. The PRS incidence is 29/39 (74.4%) and 31/41 (75.6%) in M and S groups, respectively, *P*-value = 0.897. Mannitol infusion during the anhepatic phase improved the PRS during LDLT unlike Shameddini et al. In contrast, they did not calculate the incidence of PRS, but they calculated the differences in MAP and CO before and after portal vein declamping, which is statistically questionable [8].

Total intraoperative UO and the anhepatic UO were increased in the M group. This is easily explained by mannitol’s diuretic effect. The increased UO in the M group did not imply preserved renal function post-LDLT [14].

Most intraoperative hemodynamic changes were comparable between the groups. The SVR was higher in the M group at 5 min after hepatic declamping and at closure time. The explanation is not clear, but this resulted in a higher CI in the **S** group at the closure time.

This is contradictory to the study of Chatterjee et al. [17] which showed a significant decrease in SVR, at 5 min and 15 min after infusion of the same mannitol dose. This difference may be explained by the specific criteria of LDLT as there was widespread use of norepinephrine, epinephrine, and furosemide which increases SVR.

The K+ and ionized Ca+2 did not show differences between both groups regarding the intraoperative electrolyte changes. However, both [Na+] and [Cl-] were significantly lower in the M group 5 min before and 5 min after portal reperfusion. Those changes were transient. This could be interpreted by mannitol’s volume increase effects which resulted in the dilution of both Na+ and Cl- [14]. Osmolality changes due to mannitol may also result in Na+ and Cl- loss to compensate for the increased osmolality [18].

The relative constant ionized [Ca+2] and [K+] in our study may be due to the close monitoring, continuous infusion, and correction of calcium chloride and potassium throughout the operation, especially around the reperfusion phase.

The pH, serum lactate, and LDH in the first two ICU days were similar between both groups regarding the liver graft function. Postoperative INR and albumin did not show differences except for albumin at 3 months post-LT, where albumin was statistically higher in the mannitol group. However, this difference is not clinically different as the average albumin level was in the normal range in both groups, 4.44 g/dl in the M group vs. 4.21 g/dl in the **S** group.

Liver enzymes (AST and ALT), total bilirubin, and GGT did not show differences between both groups except serum bilirubin on the 7th postoperative day. The 7th-day total serum bilirubin was higher in the M group with a median of 4.7 mg/dl versus 2 mg/dl in the **S** group. Nevertheless, bilirubin normalized on the 28th day and 3rd-month post-LT.

Likewise, the duration of ICU stay, the incidence of postoperative surgical complications, and the 3-month survival period were all similar between the groups.

We used serum creatinine for the diagnosis and grading of AKI despite its limitations in renal function evaluation. Muscle wasting and ascites besides hyperbilirubinemia overestimate the S.Cr measurement [19, 20].

Some authors suggested a corrected S.Cr formula to compensate for the acute fluid change overload in the intraoperative and intensive care period [21]. However, this formula was not validated, especially in liver transplant or cirrhotic patients. Furthermore, guidelines still consider the S.Cr as the most valid marker for AKI [22–24].

The definition of AKI in our study follows the International Club of Ascites revised definition of KDIGO guidelines, defined as a 0.3 mg/dl increase in the serum creatinine (S.Cr) in the early 48 postoperative hours [11, 12]. They removed the UO criteria from the definition as cirrhotic patients may have oliguria, but still have normal renal function. The International Club of Ascites can define both early and late AKI. Early refers to the AKI within 48 h and late refers to the AKI within 7 days [11, 12].

We did not use new markers detecting AKI as all guidelines still use S.Cr as the standard marker. No specific marker has been well-validated in LT. Our study has limitations: the study was not pre-registered, but we strictly followed the pre-designed protocol, approved by the IRB and is uploaded with the manuscript; the incidence of early AKI was lower than in the pilot study was higher than in what found in the study which makes the sample size questionable. We think that the enhanced fluid management during the study period reduced the incidence of AKI in this cohort. The study sample size (80 cases) is large when compared to similar studies in LDLT; however, it was insufficient to detect mannitol effects on the renal and liver graft functions.

In conclusion, the current LDLT recipient sample was insufficient to demonstrate that pre-reperfusion 1 g/kg mannitol infusion would reduce the risk of early AKI, PRS, early postoperative graft function, or the 3-month survival period.

## Supplementary Information

**Additional file 1: Supplementary figure S1.** (A) PAOP = pulmonary artery occlusion pressure(mmHg), (B) MPAP = mean pulmonary arterial pressure (mmHg) and (C) CVP = central venous pressure (mmHg) at 6 intraoperative measurement points: (1) immediately before skin incision, (2) the beginning of the an-hepatic (portal vein clamping), (3) 5-minutes before portal reperfusion (basal), (4) at 5 min after portal unclamping, (5) 5-minutes after hepatic arterial de-clamping and (6) at the skin closure. M group = mannitol group, S group = saline group. Data are presented as mean (SD). **Suppl Table 1.** pH, serum Lactate and LDH during the first two post-transplantation days in the ICU. **Suppl Table 2.** Postoperative laboratory values of serum AST, ALT, bilirubin, albumin and INR at post-transplant obtained on day 1, 2, 7, 28 days and after 3 months.

## Data Availability

The individual participant data will be available on reasonable request with the corresponding author after the local IRB approval.
