# Supplementary material for: Mannitol for prevention of acute kidney injury after liver transplantation: a randomized controlled trial
Source: BMC Anesthesiol. 2022 Dec 19;22:393. doi: 10.1186/s12871-022-01936-7 (PMC9762035; doi:10.1186/s12871-022-01936-7)
Supplement: Supplementary file 1 — Additional file 1: Supplementary figure S1. (A) PAOP = pulmonary artery occlusion pressure(mmHg), (B) MPAP = mean pulmonary arterial pressure (mmHg) and (C) CVP = central venous pressure (mmHg) at 6 intraoperative measurement points: (1) immediately before skin incision, (2) the beginning of the an-hepatic (portal vein clamping), (3) 5-minutes before portal reperfusion (basal), (4) at 5 min after portal unclamping, (5) 5-minutes after hepatic arterial de-clamping and (6) at the skin closure. M group = mannitol group, S group = saline group. Data are presented as mean (SD). Suppl Table 1. pH, serum Lactate and LDH during the first two post-transplantation days in the ICU. Suppl Table 2. Postoperative laboratory values of serum AST, ALT, bilirubin, albumin and INR at post-transplant obtained on day 1, 2, 7, 28 days and after 3 months. [file 12871_2022_1936_MOESM1_ESM.docx]

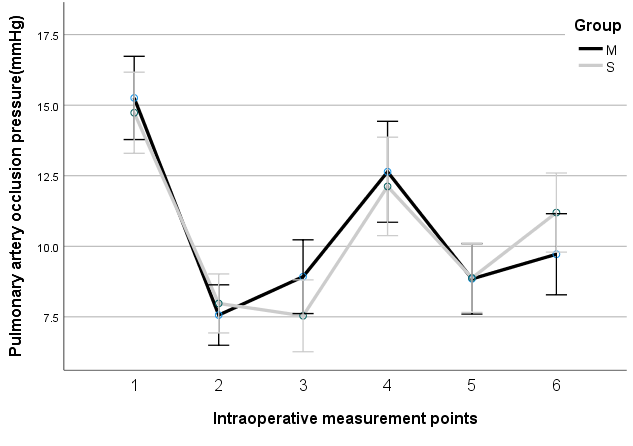

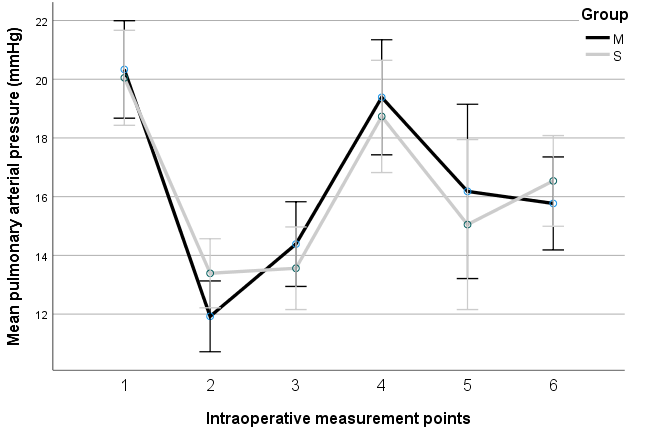


Supplementary figure S1 (A) Supplementary figure S1 (B)


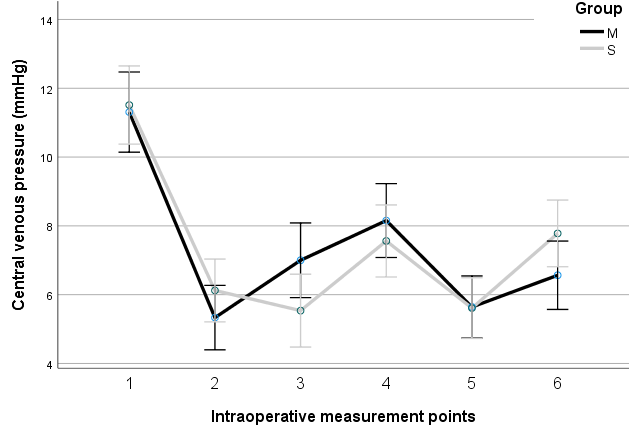


Supplementation figure S1 (C)

**Supplementary figure S1:** (A) **PAOP** = pulmonary artery occlusion pressure(mmHg), (B) **MPAP** = mean pulmonary arterial pressure (mmHg) and (C) **CVP** = central venous pressure (mmHg) at 6 intraoperative measurement points: (1) immediately before skin incision, (2) the beginning of the an-hepatic (portal vein clamping), (3) 5-minutes before portal reperfusion (basal), (4) at 5 min after portal unclamping, (5) 5-minutes after hepatic arterial de-clamping and (6) at the skin closure. **M group** = mannitol group, **S group** = saline group. Data are presented as mean (SD)

**Suppl Table 1. pH, serum Lactate and LDH during the first two post-transplantation days in the ICU**

| **Post-transplant** |  | **Group (M)**  **N = 39** | **Group (S)**  **N = 41** | ***P*-Value** |
| --- | --- | --- | --- | --- |
| **Day 1** | **pH** | 7.43 (0.046) | 7.42 (0.065) | 0.243 |
|  | **S. Lactate** (mmol/L) | 1.95 (0.58) | 2.05 (0.95) | 0.614 |
|  | **S. LDH** (U/L) | 563.03 (190.8) | 568.4 (222.6) | 0.912 |
| **Day 2** | **pH** | 7.45 (0.04) | 7.45 (0.05) | 0.84 |
|  | **S. Lactate** (mmol/L) | 1.52 (0.60) | 1.42 (0.54) | 0.424 |
|  | **S. LDH** (U/L) | 515.4 (146.1) | 450.1 (183.6) | 0.086 |

Data are presented as mean (SD). **M group** = mannitol group, **S group** = saline group

**ICU** intensive care unit, **LDH** lactate dehydrogenase

**Suppl Table 2: Postoperative laboratory values of serum AST, ALT, bilirubin, albumin and INR at post-transplant obtained on day 1, 2, 7, 28 days and after 3 months**

| **Post-transplant** |  | **Group (M)**  **N = 39** | **Group (S)**  **N = 41** | ***P*-Value** |
| --- | --- | --- | --- | --- |
| **Day 1** | **S. Cr** | 0.7 (0.6 – 1.0) | 0.7 (0.6 – 1.1) | 0.911 |
|  | **S. AST** (u/mL) | 189 (129 – 311) | 172 (106.5 – 324) | 0.56 |
|  | **S. ALT** (u/mL) | 277 (160 – 519) | 243 (140 – 509) | 0.564 |
|  | **S. total bilirubin** (mg/dL) | 2.63 (1.8) | 2.37 (1.32) | 0.391 |
|  | **S. Albumin** (g/dL) | 2.88 (0.66) | 2.97 (0.48) | 0.532 |
|  | **INR** | 1.79 (0.35) | 1.79 (0.40) | 0.957 |
| **Day 2** | **S. Cr** (u/mL) | 0.7 (0.6 – 1.1) | 0.7 (0.6 – 1.0) | 0.886 |
|  | **S. AST** (u/mL) | 121 (88 – 235) | 125 (83 – 203) | 0.641 |
|  | **S. ALT** (u/mL) | 242 (146 – 492) | 224 (135 – 427) | 0.691 |
|  | **S. total bilirubin** (mg/dL) | 2.28 (1.05) | 2.07 (0.82) | 0.339 |
|  | **S. Albumin** (g/dL) | 2.89 (0.31) | 2.85 (0.42) | 0.613 |
|  | **INR** | 1.52 (0.27) | 1.46 (0.24) | 0.24 |
| **Day 7** | **S. Cr** (u/mL) | 0.7 (0.6 – 0.9) | 0.7 (0.6 – 0.9) | 0.924 |
|  | **S. AST** (u/mL) | 33 (26 – 52) | 40 (26.25 – 53.25) | 0.586 |
|  | **S. ALT** (u/mL) | 56 (38 – 131) | 70 (46 – 107.75) | 0.891 |
|  | **S. total bilirubin** (mg/dL) | 4.7 (2.2 – 6.2) | 2 (1.3 – 3.98) | 0.007* |
|  | **S. Albumin** (g/dL) | 3.04 (0.37) | 3.08 (0.37) | 0.616 |
|  | **INR** | 1.25 (0.22) | 1.24 (0.16) | 0.83 |
| **Day 28** | **S.Cr** (u/mL) | 0.8 (0.7 – 1.15) | 0.95 (0.8 – 1.2) | 0.239 |
|  | **S. AST** (u/mL) | 21 (21- 37) | 22 (21 – 37.75) | 0.36 |
|  | **S. ALT** (u/mL) | 22 (20 – 31.5) | 21 (20 – 44.25) | 0.7 |
|  | **S. total bilirubin** (mg/dL) | 1.2 (0.75 – 1.65) | 0.85 (0.6 – 1.38) | 0.116 |
|  | **S. Albumin** (g/dL) | 3.94 (0.53) | 3.99 (0.49) | 0.645 |
|  | **INR** | 1.1 (1.0 – 1.3) | 1.0 (1.0 – 1.2) | 0.409 |
| **3 months** | **S. Cr** (u/mL) | 0.9 (0.7 – 1.0) | 0.9 (0.78 – 1.2) | 0.248 |
|  | **S. AST** (u/mL) | 32 (21 – 54) | 24 (21 – 35) | 0.147 |
|  | **S. ALT** (u/mL) | 31 (21 – 54.75) | 24.5 (20.75 – 40) | 0.381 |
|  | **S. total bilirubin** (mg/dL) | 0.65 (0.5 – 0.98) | 0.7 (0.5 – 0.83) | 0.857 |
|  | **S. Albumin** (g/dL) | 4.44 (0.43) | 4.21 (0.43) | 0.028* |
|  | **INR** |  |  | 0.061 |

Data are presented as mean (SD) or median (IQR). * Indicates statistical significance (P value < 0.05)

**M group** = mannitol group, **S group** = saline group. ***S.*** serum, **Cr**. Creatinine, ***AST*** aspartate transaminase, ***ALT*** alanine transaminase, ***INR*** International normalized ratio
